# Association between pro- and anti-inflammatory cytokines and depressive symptoms in patients with diabetes—potential differences by diabetes type and depression scores

**DOI:** 10.1038/s41398-017-0009-2

**Published:** 2018-03-09

**Authors:** Christian Herder, Andreas Schmitt, Florian Budden, André Reimer, Bernhard Kulzer, Michael Roden, Thomas Haak, Norbert Hermanns

**Affiliations:** 1 Institute for Clinical Diabetology, German Diabetes Center, Leibniz Center for Diabetes Research at Heinrich Heine University Düsseldorf, Düsseldorf, Germany; 2 German Center for Diabetes Research (DZD), München-Neuherberg, Germany; 3 Research Institute of the Diabetes Academy Mergentheim (FIDAM), Bad Mergentheim, Germany; 4 Department for Psychology, Otto Friedrich University of Bamberg, Bamberg, Germany; 5 Division of Endocrinology and Diabetology, Medical Faculty, Heinrich Heine University Düsseldorf, Düsseldorf, Germany

## Abstract

Subclinical inflammation has been implicated in the development of depression, a common comorbidity of type 1 diabetes (T1D) and type 2 diabetes (T2D). This study aimed to characterise the relationships between biomarkers of inflammation and depressive symptoms in T1D and T2D. Biomarkers of inflammation were measured in serum of participants with elevated depressive symptoms and T1D (*n* = 389, mean age 38 years, diabetes duration 15 ± 11 years) or T2D (*n* = 204, mean age 56 years, diabetes duration 13 ± 8 years). Subclinical depression was examined using three questionnaires (Center for Epidemiologic Studies Depression [CES-D], Patient Health Questionnaire-9 [PHQ-9], 5-item World Health Organization Well-Being Index [WHO-5]). In T1D, levels of interleukin-1 receptor antagonist (IL-1RA) were positively associated with two depression scores (CES-D, PHQ-9), and high-sensitivity C-reactive protein (hsCRP) was positively associated with depression for one score (WHO-5) after adjustment for age, sex, body mass index, diabetes duration, metabolic variables, medication and comorbidities (*P* = 0.008-0.042). In T2D, IL-18 and IL-1RA were positively associated with depression for two scores (IL-18: PHQ-9, WHO-5; IL-1RA: CES-D, WHO-5), hsCRP was associated with one depression score (PHQ-9), and adiponectin showed an inverse association with one depression score (PHQ-9) after adjustment (*P* = 0.006–0.048). No associations were found for IL-6 and CC-chemokine ligand 2 (CCL2). In conclusion, we observed associations between hsCRP, IL-1RA and depressive symptoms in patients with diabetes. In T2D, there was additional evidence for associations of IL-18 and (inversely) adiponectin with depressive symptoms. The strength of the associations appeared to depend on diabetes type and the method used to asssess depressive symptoms.

## Introduction

Depression is one of the most frequent comorbidities in both patients with type 1 diabetes (T1D) and type 2 diabetes (T2D)^1–4^. The high prevalence of depressive disorders in patients with diabetes represents an important clinical problem^5^ because not only major depression but also lower levels of depressive symptoms are linked with poorer long-term prognosis (e.g., increased risk of diabetes-related micro- and macrovascular complications) and higher mortality^6–10^.

Subclinical inflammation has been implicated as important pathomechanism in patients with the double burden of diabetes and depression^11–14^. Inflammatory processes contribute to the development of diabetes, although it is important to note that different types of immune activation are involved in the aetiology and pathomechanisms leading to T1D and T2D^15,16^. In addition, several lines of evidence have also linked inflammation and depression^12,14,17–19^. These include mechanistic studies in animals and clinical studies, but also cross-sectional and prospective epidemiological studies showing that high systemic levels of cytokines and chemokines are associated with a higher prevalence or risk of subclinical and major depression^20–22^.

Most of these latter studies are based on samples from the general population, whereas only few studies addressed the association between biomarkers of inflammation and depression specifically in patients with T1D^23,24^ or T2D^23–27^. Therefore, it is currently not well understood (i) if subclinical inflammation is associated with depressive symptoms in patients with diabetes, (ii) if associations differ by diabetes type and (iii) to what extent associations are independent from confounders such as glycaemic control, diabetes duration, medication use or diabetes-related comorbidities. In addition, the aforementioned studies used different methods to diagnose depressive symptoms, which might have influenced study results.

Thus, the major aim of this study was to test the hypothesis that biomarkers of subclinical inflammation are associated with depressive symptoms in a large sample of individuals with T1D and T2D. We used three different questionnaire-based scores to assess depressive symptoms, so that we were able to compare such associations not only between diabetes types, but also between depression measurements.

## Study population and methods

### Study population

This study used baseline data from two single centre randomised clinical trials with comparable study populations which were conducted at the Diabetes Center Mergentheim (a specialised inpatient diabetes care centre) and tested interventions for depressive symptoms in patients with diabetes (ClinicalTrials.gov Identifiers NCT01009138 and NCT01812291). Both studies were approved by the Ethics Committee of the State Medical Chamber of Baden-Württemberg, Germany (DIAMOS: 2009-034-f, ECCE-HOMO: F-2013-011), and performed according to the Declaration of Helsinki. All participants provided written informed consent.

The first study tested a self-management-oriented group programme (DIAMOS (Strengthening Diabetes Motivation)) which used cognitive behavioural interventions aiming at the reduction of diabetes distress. As described, patients were recruited at an inpatient diabetes centre with the following inclusion criteria: diabetes mellitus, elevated depressive symptoms (Center for Epidemiologic Studies Depression (CES-D] score) ≥ 16), age 18–70 years and sufficient German language skills. Exclusion criteria were major depression, schizophrenia/psychotic disorder, severe eating disorder, bipolar disorder, addictive disorder or personality disorder, current use of antidepressant medication or ongoing psychotherapy, being bedridden, or being under guardianship^28^.

The second study aimed to test the efficacy of a step-care approach to manage depression in diabetic patients and putative inflammatory mechanisms between diabetes and depression (acronym: ECCE-HOMO). Inclusion criteria were diabetes mellitus, elevated depressive symptoms (CES-D > 16) and/or elevated diabetes-related distress (Problem Areas in Diabetes Questionnaire (PAID) > 40), age 18–70 years, and sufficient German language skills. Exclusion criteria comprised severe clinical depression according to ICD-10 (F32.2/F32.3), suicidal ideation, severe somatic illness (e.g., end-stage diabetes complication, terminal stage cancer) or dementia. Further exclusion criteria were current psychotherapeutic/psychiatric treatment, current antidepressive medication, schizophrenia/psychotic disorder, severe eating disorder, bipolar disorder, addictive disorder, personality disorder, being bedridden, or being under guardianship.

### Assessment of depressive symptoms

Patients underwent a psychiatric interview (Composite International Diagnostic Interview/CIDI)^29^ to exclude patients with severe comorbid mental diseases, bipolar depressive disorders and major depression with suicidal ideation. Depressive symptoms were assessed by using three validated questionnaires which were filled in by the study participants. The questionnaires were given to the patients in the order listed below by an experienced clinical psychologist with precise instructions to the patients and with the opportunity to clarify any questions. After informed consent patients completed the questionnaires.

The baseline examinations of the DIAMOS and ECCE-HOMO studies included the following questionnaires to assess depressive symptoms and well-being:

- The German version of the CES-D^30,31^, a sensitive instrument to detect depressive symptoms within the previous week and to monitor changes over time;^32,33^
- The Patient Health Questionnaire-9 (PHQ-9), a depression scale to assess the frequency of each of the nine symptoms of major depression as defined by the DSM-V, which is also able to make a criteria-based diagnosis of major depression^34^, and
- The 5-item World Health Organization Well-Being Index (WHO-5) to assess subjective psychological well-being with adequate validity both as a screening tool for depression and as an outcome measure in clinical trials^35^.

CES-D and PHQ-9 scores range from 0–60 to 0–27 points, respectively, with higher scores indicating higher depressive symptoms. The WHO-5 score ranges from 0 to 25 with lower scores indicating lower well-being and more depressive symptoms. For all three questionnaires, we used the continuous scores to make optimal use of the variation in symptoms.

### Measurement of biomarkers of subclinical inflammation

Fasting blood samples were taken between 0630 and 0800 hours according to standard operating procedures on the next working day following the day when depressive symptoms were assessed. Biomarkers of subclinical inflammation were measured in serum samples at the German Diabetes Center as described^28,36^. The same assays and control sera were used for samples from both study populations. Fasting serum levels of hsCRP were measured on a Roche/Hitachi c 311 analyzer (Basel, Switzerland). Fasting serum levels of IL-6, IL-1RA, CC-chemokine ligand 2 (CCL2, also known as monocyte chemotactic protein-1/MCP-1) and total adiponectin were determined using Quantikine HS (IL-6) or Quantikine (IL-1RA, MCP-1, adiponectin) ELISA kits from R&D systems (Wiesbaden, Germany). Serum IL-18 was measured using the ELISA kit from MBL (Nagoya, Japan). These six biomarkers were chosen because of their associations with depressive symptoms and/or depression in the general population^20–27,37–39^ and their additional relationship with other diabetic comorbidities and complications^40–45^.

### Assessment of confounding variables

Demographic, anthropometric and clinical data were available from detailed baseline examinations of both trials^28^.

Information about medication was obtained from medical records. Lipid-lowering drugs included atorvastatin, fluvastatin, pitavastatin, lovastatin, pravastatin, rosuvastatin, simvastatin and ezetimibe. Non-steroidal anti-inflammatory drugs (NSAIDs) included diclofenac, ibuprofen and naproxen. Antithrombotic medication included clopidogrel, phenprocoumon, enoxaparin and aspirin. Antidepressant medication included selective serotonin reuptake inhibitors, serotonin-norepinephrine reuptake inhibitors, tricyclic antidepressants, monoamine oxidase inhibitors, reversible monoamine oxidase A inhibitors, tetracyclic antidepressants, noradrenergic and specific serotonergic antidepressant and St John’s wort.

The diagnosis of diabetic nephropathy was based on a measured glomerular filtration rate of <60 ml/min/1.73 m². Diabetic retinopathy was diagnosed by ophthalmologic examination or based on previous laser coagulation treatment. Diagnoses of cardiovascular disease, myocardial infarction, stroke or peripheral arterial occusive disease (PAOD) were made based on previous events or revascularisation measures.

### Statistical analysis

Analyses were based on 389 individuals with T1D and 204 individuals with T2D with a complete core data set comprising age, sex, body mass index (BMI), diabetes type, diabetes duration, comorbidities and medication (DIAMOS, *n* = 223 T1D and *n* = 114 T2D; ECCE-HOMO, *n* = 166 T1D and *n* = 90 T2D). Data from both studies were pooled, and analyses were then stratified by diabetes type because of well-known differences in the pathophysiology including levels of biomarkers of subclinical inflammation between T1D and T2D.

Data are presented as mean (SD), median (25th and 75th percentiles) or percentages (%) for continuous or categorical variables. Differences between diabetes types were tested using Student’s *t*-test (two-sided) or Pearson’s Chi^2^ test. Unadjusted correlations between biomarkers of subclinical inflammation (ln-transformed to approximate normal distribution) and/or depression scores were estimated using Pearson’s correlation coefficients r and corresponding *P* values. Multivariate associations between biomarkers of subclinical inflammation (independent variables, separate models for ln-transformed serum levels of each biomarker) and depression scores as outcome (dependent variables, separate models for each score) were estimated using multivariable linear regression models with increasing complexity: model 1: adjusted for age, sex and study; model 2, model 1 + adjustment for BMI, haemoglobin A1c (HbA1c), time since diagnosis of diabetes, total cholesterol, triglycerides, use of lipid-lowering drugs, hypertension, use of NSAIDs, use of antithrombotic medication, use of antidepressant medication; model 3: model 2 + adjustment for number of microvascular comorbidities and number of macrovascular comorbidities. Adjustment for the time of blood sampling was not necessary given the narrow time-window in which blood samples were taken. Regression coefficients β are standardised to 1SD of ln-transformed serum levels of biomarkers of subclinical inflammation.

In the meta-analysis from Howren et al.^20^ effect sizes of the associations between biomarkers of inflammation and depression ranged between *d* = 0.15 and *d* = 0.35. The sample size of 204 participants with T2D and 389 participants with T1D allowed the detection of significant associations between biomarkers of inflammation and depressive symptoms of a small to moderate effect size of *d* = 0.20 with a power of 82% in people with T2D and of 97% in people with T1D.

We considered *P* values of < 0.05 to indicate statistically significant differences or associations. All analyses were performed using Systat 12.0 and SPSS 23.

## Results

### Study population

The characteristics of the study population stratified by diabetes type are summarised in Table 1. Patients with T1D were younger, but had a longer time since the diagnosis of diabetes than patients with T2D. In addition, they were more often female, had lower levels of BMI, HbA1c and triglycerides than patients with T2D, whereas no group difference was found for total cholesterol. Patients with T1D were also less frequently hypertensive, less likely to use lipid-lowering and antithrombotic drugs and suffered less often from diabetes-related comorbidities compared to patients with T2D. No differences were observed between both groups regarding their use of NSAIDs and their CES-D, PHQ-9 and WHO-5 scores, but patients with T2D showed a slightly higher prevalence of antidepressant drug use. Serum levels of hsCRP, IL-6, IL-18, CCL2 and IL-1RA were lower, whereas adiponectin was higher in T1D than in T2D.

**Table 1:** Study population stratified by diabetes type

*Variable*	*T1D*	*n*	*T2D*	*n*	*P*
Age (years)	38.1 (13.1)	389	55.8 (9.1)	204	<0.001
Sex (men/women) (%)	41.6/58.4	389	56.9/43.1	204	<0.001
BMI (kg/m²)	26.0 (4.6)	389	35.3 (6.6)	204	<0.001
HbA1c (%)	8.2 (7.4; 9.3)	387	8.9 (8.0; 10.3)	202	<0.001
HbA1c (mmol/mol)	66 (57; 78)	387	74 (64; 89)	202	<0.001
Time since diagnosis of diabetes (years)	15.2 (11.2)	389	12.6 (7.6)	204	0.001
Total cholesterol (mg/dl)	190 (173; 226)	386	186 (161; 225)	202	0.895
Triglycerides (mg/dl)	93 (71; 138)	386	180 (130; 273)	202	<0.001
Lipid-lowering drugs (%)	11.8	389	45.6	204	<0.001
Hypertension (%)	23.9	389	75.0	204	<0.001
NSAIDs (%)	2.1	389	2.9	204	0.524
Antithrombotic drugs (%)	5.9	389	42.6	204	<0.001
Number of diabetes-related comorbidities	0.5 (0.8)	389	1.5 (1.4)	204	<0.001
Retinopathy (%)	20.6	389	25.5	204	0.182
Nephropathy (%)	4.1	389	18.6	204	<0.001
Polyneuropathy (%)	15.2	389	56.4	204	<0.001
Diabetic foot (%)	1.0	389	6.9	204	0.002
PAOD (%)	1.6	389	11.0	204	<0.001
Coronary heart disease (%)	1.5	389	18.1	204	<0.001
Myocardial infarction (%)	0.5	389	6.9	204	0.001
Stroke (%)	1.0	389	4.9	204	0.016
CES-D	21.0 (10.8)	379	21.5 (10.6)	196	0.568
PHQ-9	8.9 (5.0)	380	9.3 (5.2)	197	0.317
WHO-5	10.2 (5.7)	379	11.0 (6.0)	197	0.170
Antidepressant drugs (%)	1.3	389	4.4	204	0.045
hsCRP (mg/dl)	0.11 (0.06; 0.28)	376	0.30 (0.13; 0.73)	195	<0.001
IL-6 (pg/ml)	1.0 (0.7; 1.7)	376	2.4 (1.5; 3.6)	195	<0.001
IL-18 (pg/ml)	219 (178; 280)	376	274 (203; 354)	195	<0.001
CCL2 (pg/ml)	488 (376; 650)	376	529 (423; 766)	195	0.001
IL-1RA (pg/ml)	351 (264; 504)	376	727 (478; 1160)	195	<0.001
Adiponectin (ng/ml)	9879 (6251; 15,392)	376	3927 (2635; 5954)	195	<0.001

*BMI* body mass index, *CCL2* CC-chemokine ligand 2, *CES-D* Center for Epidemiologic Studies Depression Scale, *hsCRP* high-sensitivity C-reactive protein, *IL* interleukin, *IL-1RA* IL-1 receptor antagonist, *NSAIDs* non-steroidal anti-inflammatory drugs, *PAOD* peripheral arterial occlusive disease, *PHQ-9* Patient Health Questionnaire, *T1D* type 1 diabetes, *T2D* type 2 diabetes, *WHO-5* 5-item World Health Organization Well-Being Index

Continuous data are given as mean (SD) or median (25th percentile; 75th percentile), categorial variables are given as percentages (%)

Diabetes-related comorbidities include retinopathy, nephropathy, polyneuropathy, diabetic foot, PAOD, coronary heart disease, myocardial infarction and stroke (max. 8)

As shown in Table 2, the pairwise correlations between the CES-D, PHQ-9 and WHO-5 scores were similar among patients with T1D and T2D with correlation coefficients |r| between 0.70 and 0.81 (all *P* < 0.001). Positive pairwise correlations of the biomarkers of inflammation were mainly observed for hsCRP, IL-6, IL-18 and IL-1RA with stronger associations in T2D (r between 0.33 and 0.61, all *P* < 0.001) than in T1D (r between 0.07 and 0.38, *P* between < 0.001 and 0.155) (Table 2). Correlations between CCL2 and the aforementioned biomarkers were less pronounced in both diabetes types. Adiponectin levels showed inverse associations with hsCRP, IL-6 and IL-1RA in T1D (all *P* < 0.01), whereas no significant correlations were found in T2D.

**Table 2:** Univariate correlations between biomarkers of subclinical inflammation and depression scores in individuals with T1D (upper right) and T2D (lower left)

Variable	CES-D	PHQ-9	WHO-5	hsCRP	IL-6	IL-18	CCL2	IL-1RA	Adiponectin
*CES-D*	–	0.806***	−0.750***	0.043	0.051	−0.035	0.138**	0.160**	0.051
*PHQ-9*	0.811***	–	−0.703***	0.069	0.072	0.009	0.056	0.144**	0.101
*WHO-5*	−0.712***	−0.701***	–	−0.141**	−0.071	0.043	−0.087	−0.138**	−0.082
*hsCRP*	0.192**	0.176*	−0.176*	–	0.377***	0.094	−0.049	0.384***	−0.135**
*IL-6*	0.036	0.009	−0.011	0.606***	–	0.076	0.207***	0.375***	−0.163**
*IL-18*	0.046	0.064	−0.130	0.403***	0.334***	–	−0.115*	0.074	−0.076
*CCL2*	0.130	0.098	−0.085	0.049	0.198**	0.015	–	0.104*	0.033
*IL-1RA*	0.212**	0.150*	−0.233**	0.440***	0.450***	0.348***	0.249***	–	−0.303***
*Adiponectin*	−0.102	−0.134	0.065	−0.064	−0.093	−0.088	−0.019	−0.115	–

Data are given as Pearson correlation coefficients *r*. *P < 0.05; **P < 0.01; ***P < 0.001. Circulating levels of biomarkers of subclinical inflammation were ln-transformed

### Associations between biomarkers of inflammation and depressive symptoms in patients with T1D

Unadjusted analyses revealed positive correlations between biomarkers of inflammation and depressive symptom scores for hsCRP (WHO-5), CCL2 (CES-D) and IL-1RA (CES-D, PHQ-9, WHO-5) in patients with T1D (Table 2). Please note that a high level of depressive symptoms is indicated by high CES-D and PHQ-9 scores, but low WHO-5 scores, so that inverse correlations between biomarkers and WHO-5 correspond to positive correlations between biomarkers and CES-D or PHQ-9.

The associations between hsCRP and the WHO-5 score and between IL-1RA and all three depression scores persisted after adjustment for age, sex and study cohort (Table 3, model 1). After further adjustment for BMI, metabolic variables, medication use and diabetes-related comorbidities, hsCRP remained associated with the WHO-5 score, and IL-1RA remained associated with the CES-D and PHQ-9 scores (Table 3, models 2 and 3).

**Table 3:** Associations between biomarkers of subclinical inflammation and depression scores in individuals with T1D

Biomarker	Model	CES-D		PHQ-9		WHO-5	
		*β*	*P*	*β*	*P*	*β*	*P*
*hsCRP*	**1**	0.015	0.769	0.038	0.470	−**0.113**	**0.030**
	**2**	0.029	0.627	0.046	0.456	−**0.129**	**0.034**
	**3**	0.025	0.684	0.044	0.471	−**0.123**	**0.042**
*IL-6*	**1**	0.035	0.504	0.072	0.174	−0.064	0.220
	**2**	0.026	0.636	0.066	0.235	−0.054	0.325
	**3**	0.022	0.686	0.068	0.225	−0.049	0.374
*IL-18*	**1**	0.027	0.601	0.062	0.230	−0.017	0.741
	**2**	−0.002	0.966	0.035	0.512	0.016	0.763
	**3**	−0.007	0.893	0.029	0.588	0.020	0.701
*CCL2*	**1**	0.030	0.570	0.006	0.906	0.007	0.894
	**2**	0.036	0.491	−0.001	0.985	−0.001	0.978
	**3**	0.032	0.547	−0.003	0.951	0.003	0.953
*IL-1RA*	**1**	**0.140**	**0.006**	**0.131**	**0.011**	−**0.129**	**0.012**
	**2**	**0.172**	**0.004**	**0.157**	**0.011**	−0.111	0.068
	**3**	**0.163**	**0.008**	**0.152**	**0.014**	−0.100	0.104
*Adiponectin*	**1**	0.020	0.716	0.053	0.340	−0.038	0.490
	**2**	0.011	0.861	0.056	0.362	−0.062	0.307
	**3**	0.010	0.873	0.063	0.313	−0.060	0.326

Data are given as standardised regression coefficients β from linear regression analyses and corresponding *P* values. Bold print indicates nominally significant associations with *P* < 0.05. Circulating levels of biomarkers of subclinical inflammation (measured in the units as listed in Table 1) entered the models as ln-transformed variables.

Model 1: adjusted for age, sex and study.

Model 2: model 1 + BMI, HbA1c, time since diagnosis of diabetes, total cholesterol, triglycerides, use of lipid-lowering drugs, hypertension, use of NSAIDs, use of antithrombotic medication, use of antidepressant medication.

Model 3: model 2 + number of diabetes-related comorbidities.

No significant associations were observed between IL-6, IL-18 and adiponectin and any of the three scores at all levels of adjustment (Tables 2 and 3).

### Associations between biomarkers of inflammation and depressive symptoms in patients with T2D

In patients with T2D, hsCRP and IL-1RA levels were positively correlated with depressive symptoms assessed by all three scores in the unadjusted analyses, whereas no significant correlations were found for IL-6, IL-18, CCL2 and adiponectin (Table 2).

Adjustment for age, sex and the other covariables in models 1–3 strengthened some of the aforementioned associations, whereas others were attenuated (Table 4). In the fully adjusted model 3, high levels of hsCRP were associated with depressive symptoms in one score (PHQ-9), IL-18 with two scores (PHQ-9, WHO-5), IL-1RA also with two scores (CES-D, WHO-5) and adiponectin with one score (PHQ-9). IL-6 and CCL2 levels were not related to depressive symptoms in any model.

**Table 4:** Associations between biomarkers of subclinical inflammation and depression scores in individuals with T2D

Biomarker	Model	CES-D		PHQ-9		WHO-5	
		*β*	*P*	*β*	*P*	*β*	*P*
*hsCRP*	**1**	0.122	0.090	0.113	0.121	−0.116	0.121
	**2**	0.115	0.165	0.163	0.051	−0.071	0.401
	**3**	0.116	0.163	**0.165**	**0.048**	−0.071	0.402
*IL-6*	**1**	0.035	0.614	−0.002	0.973	−0.009	0.907
	**2**	0.032	0.670	0.036	0.634	0.010	0.900
	**3**	0.033	0.663	0.038	0.610	0.011	0.884
*IL-18*	**1**	0.095	0.164	0.107	0.122	−**0.161**	**0.022**
	**2**	0.115	0.107	**0.166**	**0.020**	−**0.197**	**0.007**
	**3**	0.113	0.113	**0.163**	**0.023**	−**0.196**	**0.007**
*CCL2*	**1**	−0.043	0.536	−0.071	0.315	0.015	0.841
	**2**	−0.031	0.667	−0.057	0.425	0.012	0.872
	**3**	−0.031	0.670	−0.055	0.445	0.016	0.832
*IL-1RA*	**1**	**0.162**	**0.021**	0.093	0.191	−**0.203**	**0.005**
	**2**	**0.168**	**0.041**	0.152	0.068	−**0.223**	**0.006**
	**3**	**0.168**	**0.043**	0.152	0.069	−**0.230**	**0.006**
*Adiponectin*	**1**	−0.098	0.187	−**0.158**	**0.034**	0.061	0.429
	**2**	−0.111	0.153	−**0.209**	**0.007**	0.096	0.230
	**3**	−0.109	0.164	−**0.204**	**0.009**	0.098	0.222

Data are given as standardised regression coefficients β from linear regression analyses and corresponding *P* values. Bold print indicates nominally significant associations with *P* < 0.05. Circulating levels of biomarkers of subclinical inflammation (measured in the units as listed in Table 1) entered the models as ln-transformed variables.

Model 1: adjusted for age, sex and study.

Model 2: model 1 + BMI, HbA1c, time since diagnosis of diabetes, total cholesterol, triglycerides, use of lipid-lowering drugs, hypertension, use of NSAIDs, use of antithrombotic medication, use of antidepressant medication.

Model 3: model 2 + number of diabetes-related comorbidities.

## Discussion

Our study revealed several novel associations between biomarkers of subclinical inflammation and depressive symptoms in patients with diabetes. In T1D, higher levels of hsCRP and IL-1RA were associated with more pronounced depressive symptoms. In T2D, higher levels of hsCRP, IL-18 and IL-1RA and lower adiponectin levels were associated with higher depressive symptoms. These associations were independent of multiple confounders, but differed in their strength by the measurements that were used to assess depressive symptoms.

### hsCRP, IL-6 and depressive symptoms

The acute-phase protein CRP and the proinflammatory cytokine IL-6 are the most frequently investigated biomarkers of inflammation in cross-sectional and prospective studies on depression. Several meta-analyses demonstrated positive associations between both proteins, depressive symptoms and major depression in community- or population-based samples^20,21,37^.

This study found associations between higher hsCRP and depressive symptoms assessed by one of the three scores in patients with T1D (WHO-5) and T2D (PHQ-9). The relationship between hsCRP and depressive symptoms has been analysed before in children and adolescents with T1D in the USA^23^. and in adult patients with recent-onset T1D in Germany^24^, but results were conflicting. A recent report linking higher levels of hsCRP with a poorer response to depression treatment in patients with T1D argued in favour of clinical relevance of hsCRP in patients with depression and T1D^46^. For T2D our results confirm findings from other patient samples^23–25,27^. Collectively, it appears that associations between hsCRP and depressive symptoms are similar in individuals with and without diabetes and may be independent of diabetes type.

In contrast, we observed no associations between IL-6 levels and depressive symptoms in patients with T1D or T2D. This finding is mainly in line with previous studies in diabetes patients^23,24,27^. Thus, our study corroborates the notion that IL-6 may not be associated with depressive symptoms in patients with diabetes and that diabetes may have an effect-modifying impact on the association seen in the general population^20,37^. Given the strong correlations between hsCRP and IL-6 levels this result appears surprising, and so far it is unclear which mechanisms may be responsible for this observation.

### IL-18, IL-1RA and depressive symptoms

IL-18 and IL-1RA are members of the IL-1 cytokine family. IL-18 has proinflammatory activities, whereas IL-1RA is an counterregulator of the potent proinflammatory cytokine IL-1β^40^. IL-1β and IL-18 are released upon activation of the Nod-like receptor pyrin containing 3 (NLRP3) inflammasome. The activation of the NLRP3 inflammasome affects immune cells, insulin-responsive cells including pancreatic beta cells and cells within the central nervous system. Thus, it has not only been implicated in the development of T1D and T2D^40,41^, but has also been found in chronic stress models and patients with major depressive disorder^19,42^. Therefore, associations between IL-1 cytokine family members, diabetes and depressive symptoms are biologically plausible.

This study indeed found an association between higher IL-18 levels and depressive symptoms in T2D, but not in T1D. The positive association for IL-18 was observed for the PHQ-9 and WHO-5 scores, which have not been used before in relation to this cytokine in patients with T2D. The association failed to reach significance for CES-D as measure of depression, which is in line with the null finding of our previous study in patients with recent-onset T2D, which was also based on the CES-D score^24^. Our null findings for patients with T1D corroborate two previous reports^23,24^. Therefore, this study suggests a difference between T1D and T2D with respect to the association of IL-18 with depressive symptoms, but it was not adequately powered to investigate such a difference with a formal interaction test. However, the strong correlations between IL-18 and hsCRP, IL-6 and IL-1RA in patients with T2D in contrast to their absence in T1D point towards differences in the regulation and/or the pathophysiological relevance of this cytokine between both diabetes types. The finding of a positive association of IL-18 with depressive symptoms in T2D is interesting, because this cytokine is also involved in the development of other diabetes-related comorbidities^43,44^.

A comparison with IL-18 data from the general population is difficult because a recent meta-analysis was inconclusive due to the overall low number and sample sizes of available studies and the high heterogeneity between studies^38^. However, there is increasing experimental evidence linking stress, IL-18 and depression and in a complex manner. Induction of sadness increased circulating IL-18 levels in individuals without and with depression, possibly mediated by µ-opioid system activation, while neutral mood induction reduced IL-18^47–49^. Based on these data, higher IL-18 levels could be interpreted as marker for dysregulated emotion. Studies in mice demonstrated that knockout of IL-18 showed reduced depressive-like behaviour^50^ and that the induction of IL-18 in the basolateral amygdala by chronic stress resulted in depressive-like behaviour, which could be suppressed by local inhibition of the IL-18 system in the amygdala^51^. Thus, IL-18 may play an important role in linking environmental stimuli and the development of depressive symptoms.

Our study also demonstrated that higher IL-1RA levels were associated with depressive symptoms in both T1D and T2D. The data for T1D are novel, because we are not aware of other studies which analysed IL-1RA in this context. Our results for T2D are in line with a study in patients from the UK^27^, with a meta-analysis of community-based or population-based studies^20,52^ and with a prospective study showing that higher IL-1RA levels were related to higher risk of developing depressed mood in older persons^53^. Thus, our study extends the evidence that systemic IL-1RA levels are upregulated in patients with depressive symptoms to patients with T1D. The difficulty to reliably measure circulating levels of IL-1α and IL-1β means that we could not investigate the ratio between these cytokines and IL-1RA as antagonist. IL-1α acts mainly as intracellular cytokine, and serum or plasma levels of IL-1β are below the levels of detection for most individuals in the absence of major trauma or infection, when high-sensitivity ELISAs as gold-standard method for protein quantification are used^40^. Nevertheless, these data indicate that IL-1-related processes and compensatory anti-inflammatory mechanisms may be relevant in patients with depression irrespectively of their diabetes status as previously also shown for macro- and microvascular comorbidities of diabetes^40,42,45^.

### CCL2, adiponectin and depressive symptoms

Data on associations between the chemokine CCL2 and the adipokine adiponectin with depression are relatively scarce. We observed no associations between CCL2 and depressive symptoms in T1D and T2D, but an inverse association between adiponectin and depressive symptoms in patients with T2D.

The data for CCL2 are novel because we are not aware of comparable analyses in patients with T1D. CCL2 levels have been found associated with depressive symptoms in one study in patients with T2D^27^, which we could not replicate. A recent meta-analysis reported higher CCL2 levels in patients with major depression compared to healthy controls. However, it also demonstrated high heterogeneity between studies and provided evidence that this result might be attributable to substantial publication bias^22^. CCL2 represents a relevant biomarker in the context of diabetes because higher systemic levels are associated with higher risk of other comorbidities and mortality^54^, but its potential relevance for depression remains to be elucidated.

An association between lower adiponectin and more pronounced depressive symptoms has not been reported in other studies in T2D^23,24,27^, whereas our null findings in T1D corroborate previous reports^23,24^. Data from a recent meta-analysis are in line with our result for T2D, because it suggests that adiponectin levels may be lower in patients with major depression compared to controls without depression. However, it also indicated that this difference may be partly explained by confounding factors^39^.

### Clinical relevance of our findings and implications for future studies

The findings for IL-6 emphasise that results may differ between individuals without and with diabetes, so that diabetes status should be investigated as potential effect modifier in future studies. The data from this study also suggest that associations between biomarkers of inflammation and depressive symptoms may be similar for some proteins (hsCRP, IL-1RA), but different for others (IL-18, adiponectin) when comparing patients with T1D and T2D. The number of prior studies that have investigated patients with both diabetes types in the same analysis is small^23,24^, and all three studies (i.e., the aforementioned studies and this one) were based on patients who varied in age and diabetes duration. Nevertheless, the currently available evidence points towards the possibility that such differences between T1D and T2D exist and argues in favour of analyses stratified by diabetes type rather than joint analyses of diabetes patients in this context.

Finally, the use of three questionnaires to assess depressive symptoms was helpful to cross-validate results, but it is important to keep in mind that different instruments measure different patterns of depressive symptoms. It seems plausible that such differences are also reflected by variations in associations between biomarkers of inflammation and these scores. We observed two significant associations of biomarkers with the CES-D score, four with the PHQ-9 and three with the WHO-5 score. Although no biomarker was associated with all three scores it is noteworthy that in cases with two significant associations the third one had only slightly lower effect sizes and *P* values between 0.069 and 0.113, indicating that the lack of statistical significance was mainly due to sample size. Taken together, our findings highlight the need for precise descriptions of depression outcomes in biomarker studies and the difficulties in comparing or meta-analysing studies using different measurement scores and/or definitions of depression.

Thus, our study may help to inform future studies seeking to gain further insights into the relationship between inflammation, diabetes and depression. Important questions for further research concern the direction of effects, the impact of immune activation on the response to depression therapy and the underlying molecular mechanisms which could be targeted. There is evidence that depression may cause inflammation and vice versa because anti-depressant therapy has been reported to decrease systemic levels of inflammation-related biomarkers^55,56^, while anti-inflammatory agents may alleviate depressive symptoms^57,58^. In addition to this potentially bidirectional relationship, both inflammation and depression likely share biological pathways^11,13,14,59^ such as the aforementioned activation of the NLRP3 inflammasome^19,42^, which adds to the complexity of the cross-talk between the immune system and the central nervous system, but may also present novel therapeutic opportunities^19,60,61^.

### Strengths and limitations

Strengths of our study are the large sample for association analyses, the inclusion of both patients with T1D and T2D, the use of three instruments to assess depressive symptoms and the measurement of six biomarkers reflecting different facets of immune activation. Additionally, the study participants were also well phenotyped with respect to potentially confounding factors such as comorbidities. Finally, we used few exclusion criteria for the study population to limit selection bias.

Limitations include the cross-sectional design and the sample size that was too limited for formal interaction tests to compare results between diabetes types or depression scores. Our focus on diabetes patients with subclinical depression and mild-to-moderate depression entailed the lack of inclusion of diabetes-free controls and depression-free controls or patients with severe major depression. The fact that our study relied on patients with diabetes and subthreshold or mild forms of depression also meant that we had a reduced variance of depression severity due to exclusion of people without depressive symptoms and those with severe depression. Our study did not contain other inflammation-related biomarkers which might also have been of interest in this context (e.g., IL-1β, IL-17, TNFα etc.), partly because of the aforementioned analytical reasons and partly because data implicating systemic levels of these biomarkers in the pathophysiology of diabetic complications are more limited than for the other selected biomarkers. However, a more comprehensive characterisation of subclinical inflammation by measurement of further biomarkers will undoubtedly contribute to a better understanding of its role for the development of depression in patients with T1D and T2D. We did not correct for multiple testing due to the strong correlations both among biomarkers and among depression scores, so that results should be considered exploratory. Due to different samples sizes of people with T1D and T2D, the statistical power to detect significant associations differed between both subsamples. Lastly, data may not be generalisable to diabetes patients with non-European descent.

## Conclusion

This study showed differential associations between biomarkers of subclinical inflammation and depressive symptoms in patients with type 1 and type 2 diabetes rather than a general immune activation in diabetic individuals with depressive symptoms. These immune profiles show overlaps with risk factors for other comorbidies of diabetes. In conclusion, the results provide preliminary evidence for disease- and/or diabetes type-specific associations which may depend on the instrument used to assess depression.
